# Time course of changes in heart rate and blood pressure variability in rats with myocardial infarction

**DOI:** 10.1590/1414-431X20165511

**Published:** 2017-01-09

**Authors:** R. Aires, E.B. Pimentel, L. Forechi, E.M. Dantas, J.G. Mill

**Affiliations:** 1 Departmento de Ciências Fisiológicas, Universidade Federal do Espírito Santo, Vitória, ES, Brasil; 2 Colegiado de Ciências Biológicas, Universidade Federal do Vale do São Francisco, Petrolina, PE, Brasil

**Keywords:** Myocardial infarction, Heart rate variability, Blood pressure variability, Autonomic nervous system, Heart failure

## Abstract

Our aim was to determine the time course of changes in autonomic balance in the acute (1 and 3 days), sub-acute (7 days) and chronic (28 days) phases of myocardial infarction (MI) in rats. Autonomic balance was assessed by temporal and spectral analyses of blood pressure variability (BPV) and heart rate variability (HRV). Pulsatile blood pressure (BP) recordings (30 min) were obtained in awake and unrestrained male Wistar rats (N = 77; 8-10 weeks old) with MI (coronary ligature) or sham operation (SO). Data are reported as means±SE. The high frequency (HF) component (n.u.) of HRV was significantly lower in MI-1- (P<0.01) and MI-3-day rats (P<0.05) than in their time-control groups (SO-1=68±4 *vs* MI-1=35.3±4.3; SO-3=71±5.8 *vs* MI-3=45.2±3.8), without differences thereafter (SO-7=69.2±4.8 *vs* MI-7=56±5.8; SO-28=73±4 *vs* MI-28=66±6.6). A sharp reduction (P<0.05) of BPV (mmHg^2^) was observed in the first week after MI (SO-1=8.55±0.80; SO-3=9.11±1.08; SO-7=7.92±1.10 *vs* MI-1=5.63±0.73; MI-3=5.93±0.30; MI-7=5.30±0.25). Normal BPV, however, was observed 4 weeks after MI (SO-28=8.60±0.66 *vs* MI-28=8.43±0.56 mmHg^2^; P>0.05). This reduction was mainly due to attenuation of the low frequency (LF) band of BPV in absolute and normalized units (SO-1=39.3±7%; SO-3=55±4.5%; SO-7=46.8±4.5%; SO-28=45.7±5%; MI-1=13±3.5%; MI-3=35±4.7%; MI-7=25±2.8%; MI-28=21.4±2.8%). The results suggest that the reduction in HRV was associated with decrease of the HF component of HRV suggesting recovery of the vagal control of heartbeats along the post-infarction healing period. The depression of BPV was more dependent on the attenuation of the LF component, which is linked to the baroreflex modulation of the autonomic balance.

## Introduction

Cardiovascular disease is the leading cause of death worldwide, with nearly 17 million deaths occurring each year. Myocardial infarction (MI) accounts for almost 7 million of these events (1). Acute MI causes a reduction in the pumping capacity of the heart, which reduces the cardiac output and blood pressure (BP). These changes are directly related to the area of the left ventricle affected by the ischemic damage (2,3).

Within the first hours after coronary occlusion, ischemic cardiac tissue undergoes cell death due to necrosis and apoptosis (2,4). Cardiac cells from the ischemic region become unable to exert their contractile function. Remodeling occurs over time. However, the restoration of mechanical pumping capacity is generally incomplete, such that progression to chronic heart failure is often the natural history of individuals who survive a large MI (2).

The sympathetic and parasympathetic inputs to the heart and to the blood vessels are closely regulated to maintain cardiovascular homeostasis within limits necessary to provide adequate blood circulation to the entire body (5). Therefore, maintenance of normal reflexes to adjust BP regulation is important for cardiovascular homeostasis (6).

In the acute phase of MI, sympathetic hyperactivity seems to be an adaptation to compensate for the reduction in cardiac mechanical performance and to maintain cardiac output and BP within normal ranges (7). The increase in sympathetic drive, however, leads to several deleterious consequences to the heart, including the facilitation of tachyarrhythmia and increased oxygen consumption in the myocardium. Therefore, several adaptive processes are activated to restore normal autonomic balance over time (8,9).

Analysis of heart rate variability (HRV) has been used to assess the autonomic balance to the cardiovascular system. The use of this technique has increased in the last decades in clinical and experimental settings because it is non-invasive and depends only on an adequate electrocardiogram recording. HRV can also be obtained by recording pulsatile BP, because the interbeat interval can be calculated from the peak values of two consecutive curves of systolic pressure. Moreover, pulsatile BP allows the determination of blood pressure variability (BPV), which is closely dependent on baroreceptor signaling to the central structures responsible for tonic control of the resistance vessels of the systemic circulation, thus interfering with BP values (5,10). HRV has been used in humans as a prognostic tool because a high sympathetic tone after infarction is a marker of poor evolution and increased mortality (11,12).

Despite evidence of gradual recovery of the normal autonomic balance in the heart after MI, few studies have investigated the time course of this process. Moreover, despite the great number of studies focusing on HRV after MI, BPV has been poorly studied in this condition. Therefore, our aim was to determine the time course of changes in autonomic balance in rats subjected to MI produced by coronary artery ligature.

## Material and Methods

### Animals

Young male Wistar rats (n=77, 8–10 weeks old, 200 to 300 g) from our institutional colony (Centro de Ciências da Saúde, Universidade Federal do Espírito Santo, Brazil) were used in all experiments, conducted in accordance with the ethical NIH guidelines for animal research (Guide for the Care and Use of Laboratory Animals, NIH Publication No. 85-23, revised 1996). The project was approved by our institutional committee on ethical animal use for research proposals (protocol #073/2013). During the experimental period, the animals had free access to water and food (Labina¯, Purina, Brazil) and were subjected to a 24-h standard circadian cycle (12-h light/dark) in a room with controlled temperature (20–24°C).

### Production of myocardial infarction

The animals were randomly divided into two groups: one underwent coronary ligation to produce MI, and the other was submitted to a sham operation (SO) surgery. Sub-groups of MI and SO rats were then studied at 1, 3, 7, or 28 days after the surgical procedure. MI was produced according to standard techniques described elsewhere (4). The animals were weighed and anesthetized with intraperitoneal injections of ketamine (30 mg/kg) and xylazine (10 mg/kg). After loss of reflexes, the animals underwent a left thoracotomy in the fourth intercostal space. The anterior descending branches of the left coronary artery were firmly tied with 6-0 mononylon thread between the left atrial border and the pulmonary outflow tract. Placed at this point, the ligature produced an infarcted area encompassing 30 to 40% of the left ventricle circumference (4). In the SO animals, the same surgical procedures were performed except for suturing the coronary vessels, which remained untied.

To avoid the interference of anesthetics on the autonomic input to the heart, a catheter was implanted 24 h prior to recording BP. Thus, the animals studied 1 day after MI or SO were submitted to cannulation of the left femoral artery just before surgery to produce MI or SO. A PE50 attached to a PE10 polyethylene catheter was inserted into the left femoral artery and tunneled subcutaneously to the dorsal region of the neck to record pulsatile BP in awake and unrestrained animals 24 h later. Sub-groups of animals were studied in the acute (1 or 3 days), sub-acute (7 days) or chronic phase (28 days) of the post-infarction period, when the scar tissue is fully consolidated in rats (3,4).

### Heart rate and blood pressure variability

The femoral catheter filled with heparin solution (1:50 IU) was connected to a pressure transducer (TRI 21 Letica Scientific Instruments, Spain) and amplified (110 ML, ADInstruments, Australia) for continuous recording of pulsatile BP for a period of 30 min (after record stabilization). BP was always recorded in the morning (8:00 to 11:00 am) in a quiet and temperature-controlled (22–24°C) room to reduce ambient stress. The analogical signal was digitally converted by a data acquisition system (PowerLab 4 SP, ADInstruments), and BP signals were recorded by Chart 5.5.1 software (Australia) using a sampling rate of 1 kHz for posterior off-line evaluation of HRV and BPV (13). The recorded pressure signals were manually pre-processed to remove ectopic beats and artifacts. Next, the software detected the peaks of the systolic waves and pulse intervals (PI) and the series of systolic blood pressure (SBP) values were generated. Then, both series were filtered with appropriate parameters for recording in rats (14), and the SBP and PI series (the latter corresponding to the RR interval of the ECG) were submitted to temporal and spectral analysis.

The PI and SBP variances were calculated in the time domain analysis. Frequency domain analysis was performed by the autoregressive method according to an algorithm developed in our laboratory (15). The time series were divided into continuous segments of 300 beats, overlapped by half. The oscillatory components were calculated by the Yule-Walker method with the Levison-Durbin recursion. The oscillatory components were divided into three bands comprising very low frequency (VLF 0.01 to 0.20 Hz), low frequency (LF 0.20 to 0.75 Hz) and high frequency (HF 0.75 to 2.50 Hz) bands expressed in ms^2^ or in mmHg^2^ for HRV and BPV analyses, respectively. The LF and HF components were also expressed in normalized units (n.u.). Normalization consisted of dividing the power of each component by the total power after subtracting the power of the VLF component. The LF/HF ratio was obtained by dividing the LF and HF components in n.u. (11).

### Morphological analysis of the heart

After hemodynamic recording, the animals were euthanized by an anesthetic overdose. The chest was quickly opened, and the heart was removed and washed in saline solution. The ventricles were separated from the atria by sectioning of the atrioventricular ring, and the free wall of the right ventricle was carefully separated from the left ventricle. Excess water was removed from the heart tissues with filter paper, and the chambers were weighed separately. The relative weight of the ventricular chambers was corrected to the body weight. Infarct size was determined by cutting the left ventricle into three transverse slices, which were immersed in 1% solution of triphenyltetrazolium chloride (Sigma, USA) in phosphate buffered saline for 10 min at 37°C.

The infarct size of the animals studied 1 or 3 days after coronary ligature is reported as the percentage of total area of the left ventricle. In the groups studied 7 or 28 days after surgical procedure, the epicardial perimeter of the left ventricle was defined manually in ImageJ software (v. 1.43 u, National Institutes of Health, USA). Thereafter, the corresponding epicardial circumference of the infarcted area was defined, and the infarct size was given as the percentage of left ventricular circumference occupied by the infarct scar (4).

### Statistical analysis

Data are reported as means±SE. A two-way analysis of variance (ANOVA) followed by Fisher's *post hoc* test was used to compare the means at different times after surgery, as well as to compare MI and SO rats examined at the same time after surgery. Differences between groups were considered significant when P<0.05.

## Results

### Infarct size, body and heart weights

No significant differences were observed in infarct size in MI rats among the groups studied at different times during the post-infarction period (Table 1). Before surgery, the initial body weights of the animals submitted to MI or SO were similar (P=0.69). A small loss of body weight was observed in the first week after surgery. However, this decrease was similar in rats submitted to coronary ligation or to sham surgery, suggesting that similar surgical trauma occurred in both groups. Table 1 shows the body weights and heart weights of MI and SO rats studied at 1, 3, 7 and 28 days after surgery. Left ventricular weight remained stable in the SO and MI groups at different times after surgery, whereas right ventricular hypertrophy was observed in rats with MI studied 28 days after coronary ligature.

**Table:**
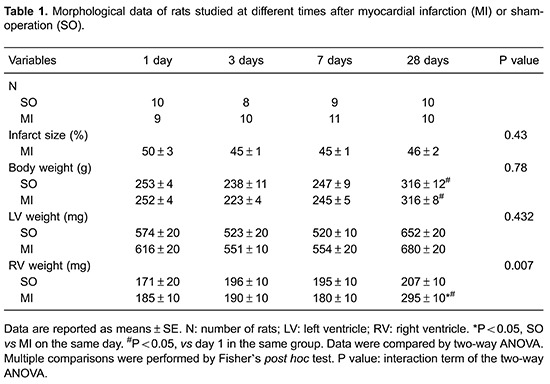

### HRV analyses

**Figure 1:**
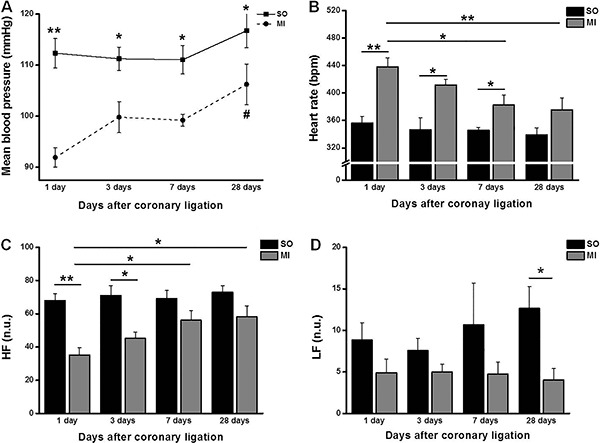
Mean blood pressure (*A*), heart rate (*B*) and high frequency (HF) (*C*), low frequency (LF) (*D*) components in normalized units (n.u.) of heart rate variability in sham operated (SO) and infarcted (MI) rats studied at different times after surgery. Results are reported as means±SE. Data were compared by two-way ANOVA and multiple comparisons were performed by the Fisher’s *post hoc* test. *P<0.05; **P<0.01; ^#^P<0.05 SO-28 *vs* MI-28.

The mean BP of rats with MI was lower 1 day after coronary ligature compared to SO rats (Figure 1A). Despite a small recovery of BP levels along the post-infarction period, the mean BP remained lower in this group along the whole follow-up period. Heart rate was stable throughout the follow-up period in SO rats. However, unlike BP, heart rate was higher at 1, 3, and 7 days after MI (Figure 1B). This increase in heart rate was progressively attenuated after MI so that no significant difference was observed in the rats studied 28 days post-coronary ligature (SO-28=339±10 *vs* MI-28=375±17 bpm; P>0.05), despite that a tendency to higher values was still present.

The spectral components of HRV (in normalized units) are shown in Figure 1 (panels C and D). The HF component, which reflected parasympathetic heartbeat modulation, was significantly reduced at 1 and 3 days after infarction. Normal HF power was progressively recovered in the groups of animals studied 7 and 28 days after coronary ligature. A consolidated scar was found in the latter group. The LF component (Figure 1D) tended to be lower in rats with MI. However, significant differences between the groups were observed only in the chronic infarct period (4 weeks after MI).

Table 2 shows the analysis of HRV in the time and frequency domains of the SO and MI animals in the post-surgery period. The total PI variance remained stable over time in the SO group, whereas in the group with coronary ligature, higher PI variance was observed when analyzing the entire study period. In the frequency domain analysis, it was observed that the power of the VLF component was predominant in both groups throughout the post-surgical period. Total absolute power of HRV was smaller in MI rats studied one day after coronary ligature (HF+LF+VLF=10.3 ms^2^). However, no significant changes were seen in the LF absolute component of HRV after MI, and no consistent pattern was seen over time. Fast recovery of HRV seems to have occurred in the MI animals due to vagal input; these changes were observed mainly in PI variance and the HF component, as well as in the sum of VLF, LF and HF power spectrum. Three days after surgery, these values were higher in rats with MI than in SO rats. The LF/HF index of HRV was not significantly changed in MI rats.

**Table:**
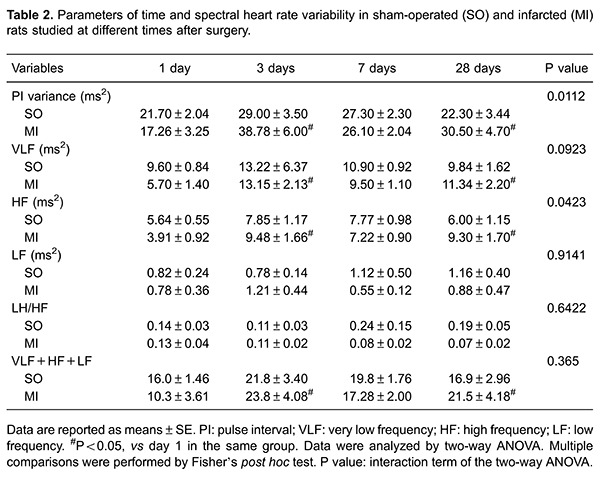

### BPV analyses

**Figure 2:**
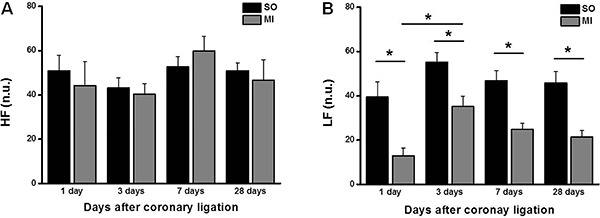
Spectral analysis of blood pressure variability. High frequency (HF) (*A*) and low frequency (LF) (*B*) components given in normalized units (n.u.) in sham-operated (SO) and infarcted (MI) rats studied at different times after surgery. Results are reported as means±SE. *P<0.05; data were compared by a two-way ANOVA and multiple comparisons were performed by Fisher’s *post hoc* test.

Table 3 shows systolic BPV in SO and MI rats at different times after surgery. A significant reduction in BP variability was observed in the infarcted animals throughout the entire post-infarction period without a tendency toward recovery. In the spectral analysis, it was observed that oscillations in the VLF component were significantly attenuated in the MI group without significant changes in the post-infarction period. The HF component in absolute (Table 3) or in normalized units (Figure 2A) remained unchanged after infarction, exerting minimal influence on total variability. The LF component, however, was attenuated in infarcted animals throughout the observation period (Figure 2B).

**Table:**
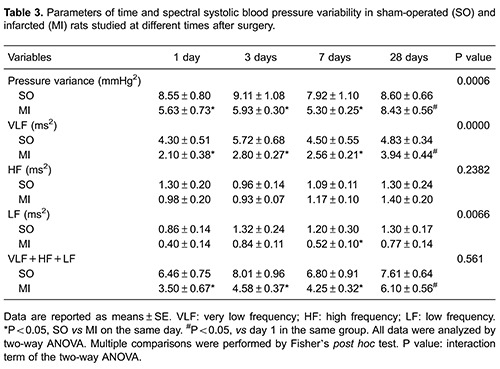

## Discussion

Autonomic balance plays a significant role in cardiovascular homeostasis because the sympathetic and vagal discharges directed to the heart and blood vessels interfere with many cardiovascular parameters, including heart rate, peripheral vascular resistance, cardiac output and BP. Moreover, autonomic balance influences the electrical stability of cardiac cells with or without automatism, thus interfering on the development of cardiac arrhythmias (7,16,17). Autonomic imbalance to the heart after infarction has been documented in animal models (18,19) and in humans (20,21). However, no study has explored simultaneous changes in heart rate and BP variability at different phases of the infarction healing process. Therefore, the present study was designed to assess hemodynamic changes after MI and to link these changes to the autonomic components regulating BP and HR variability.

Our study showed that MI globally reduces either the HRV as the systolic BPV. Most of the previous studies in this area, mainly in humans, have focused on changes in HRV. Our study showed that unlike HRV, in which the HF component is more affected, the LF component of systolic BP variability seems to be the most affected in rats with MI. The HF component of HRV, which is closely linked to vagal modulation of heartbeats, showed progressive recovery over 4 weeks of the post-infarction period, whereas the decreased values of the LF component persisted throughout the recovery period suggesting maintenance of a high sympathetic drive to the heart along the observation period.

The positive chronotropic response observed in the acute phase of coronary occlusion is usually considered to be an isolated effect of sympathetic activation (22,23). However, our data suggest that removal of parasympathetic control to the heart seems to significantly contribute to this finding, which seems to compensate for the reduction in the cardiac output after MI (2,3). Therefore, the development of tachyarrhythmias, which are more frequent in the acute infarct phase, may depend not only on sympathetic activation but also on the removal of the vagal control of the cardiac automaticity. Thus, progressive recovery of vagal modulation to the heart may inhibit the anomalous cardiac automaticity thus contributing to prevent development of tachyarrhythmias.

The rapid onset of tachycardia after infarction and its progressive attenuation during the post-infarction period were observed in previous studies (24,25). Recently, we showed that this sympathetic activation arises soon after coronary occlusion (15 to 30 min) when the ligature is placed in *in vitro* conditions in a working heart-brainstem preparation (26). After this precocious activation, our data suggest that the increased sympathetic drive to the heart is maintained along the four weeks of the post-infarction period.

Even considering that the electrocardiogram record is a safe procedure in humans, the use of animal models is still necessary to investigate the mechanisms of post-infarction adaptations because the use of drugs interfering with autonomic function is mandatory after infarction in humans. The use of beta-blockers and antagonists of the renin-angiotensin system interferes with cardiovascular autonomic modulation, making it difficult to follow temporal changes in HRV and BPV during the infarct healing. Experimental studies (27,28) show that starting from the second week post-MI, the spectral variables of BP and heart rate were not different throughout the course of the disease. For example, Lee et al. (29) evaluated autonomic variation in rats from 2–8 weeks after coronary ligation via electrocardiogram recording by telemetry. The authors found no change in spectral power over the observation period. Our study showed that at this stage, the recovery of the normal balance is occurring. The velocity of this recovery, however, may depend on the extension and localization of the infarcted area, making comparison among studies difficult.

Some studies in humans corroborate our findings. Data obtained by Kardos et al. (30) show attenuation of the power spectrum 3 to 5 days after infarction in patients compared with controls, indicating that sympathetic activation may contribute to this finding but is not solely responsible for the post-infarction tachycardia. We also observed this finding in our study in rats free from the influence of drugs that affect autonomic modulation. Lombardi et al. (31) also observed that in infarcted patients evaluated in the first few hours after coronary occlusion, the LF component of HRV was predominant compared with to the decreased HF component, suggesting a sympathetic increase and vagal withdrawal. This early activation of the sympathetic tone agrees with the data observed in *in vitro* perfused heart (26).

Previous data have shown decreased baroreflex sensitivity after infarction in rats, with a progressive recovery of the normal pattern over 8 weeks of the post-infarction period (19,24). In our study, a tendency to baroreflex recovery was observed in the group studied 4 weeks after infarction. It is likely that impairment of the baroreflex contributes to reduce the power of the LF component of HRV, a finding also observed by Moak et al. (32) in humans with depressed baroreflex function. In our study we observed low values of the LF component of BPV in rats with MI. It is possible that the total spectrum of these animals may contain components not included in the bands used in our analysis.

The origin of changes in autonomic modulation directed to the cardiovascular system after infarction is under discussion. These changes may arise either from the efferent pathways (origin in the central nervous system) or from afferent information to the central nervous system (origin in peripheral organs such as heart and blood vessels). In a previous study, we observed reduction of the bradycardic response of infarcted rats submitted to direct vagal stimulation (33), suggesting a possible desensitization of the cholinergic receptors of the heart after infarction. Baroreceptor desensitization may also exert a central role in this process because changes in the afferent information to the central nuclei are important to regulate the sympathetic and vagal tone directed to the entire cardiovascular system (6,19,24). LF power is modulated by the baroreflex thus affecting the sinus node response to BP changes (34). In rats with sinoaortic denervation (SAD), the power of the LF component also decreases compared with control animals (35,36). SAD, however, produces an acute and dramatic increase in BP lability (37). Based on these data, an increase and not a decrease in BP variability should be expected to occur after MI. SAD, however, leads to an increased variability of the BP as a consequence of body or head movements. Rats with MI show a low cardiac output and we speculate that reduced body movements in such conditions (mainly in the first week) may contribute to our findings. Decreased BPV associated to baroreceptor dysfunction was also observed in infarcted rats in a previous study (19). These authors also observed recovery of BPV in association with recovery of baroreceptor function 8 weeks after MI. Our observation period was only 4 weeks, but the partial recovery of BP variance 28 days after MI in our study is in accordance with this view.

Kardos et al. (30) observed that patients in the acute phase of infarction (3 to 5 days) show reduction in all of the spectral components of BPV compared with healthy individuals. They suggested that poor adaptation of the stroke volume of the infarcted heart is the cause of this finding. Teerlink and Clozel (28) attribute the depression of LF fluctuations in BP to a depressed ejection fraction after infarction. Therefore, the hemodynamic changes acutely produced after infarction may explain the low LF component found in our study.

The spectral VLF band of BPV has a complex origin because it is associated with neurohumoral regulation of the circulation, including the renin-angiotensin-aldosterone system and thermoregulation (38,39). In our analysis BPV variability was assessed in a small period of time. Therefore, the number of heart cycles is small to detect the origin of VLF components. Other studies have explained the reduction of this spectral component as a consequent of a reduced myogenic response in arterioles (40). Reduction of myogenic tone in peripheral blood vessels may be a compensatory response to the reduced cardiac stroke after MI. However, more adequate methods should be used to investigate the origin of VLF changes after MI.

In conclusion, our data show an overall reduction in HRV and BPV in the acute and subacute phases of infarction. The reduction in HRV was associated with the attenuation of the HF component and variance of PI, which are more dependent on vagal modulation of heart beats. The reduction of BPV was associated with depression of the LF component, which is linked to baroreflex sensitivity. Depression of the LF component was also found in rats after sinoaortic denervation (36). Since rats submitted to atropine show reduction of HRV and BPV (19), we can speculate that vagal dysfunction after MI represents a key step for our findings.
